# Gene Expression Analyses Support Fallopian Tube Epithelium as the Cell of Origin of Epithelial Ovarian Cancer

**DOI:** 10.3390/ijms140713687

**Published:** 2013-07-01

**Authors:** Daniel J. O’Shannessy, Stephen M. Jackson, Natalie C. Twine, Bryan E. Hoffman, Zoltan Dezso, Sergei I. Agoulnik, Elizabeth B. Somers

**Affiliations:** 1 Department of Translational Medicine and Diagnostics, Morphotek, Inc., 210 Welsh Pool Rd., Exton, PA 19341, USA; E-Mails: sjackson@morphotek.com (S.M.J.); bhoffman@morphotek.com (B.E.H.); esomers@morphotek.com (E.B.S.); 2 Biomarkers and Personalized Medicine Core Functional Unit, Eisai Inc., Four Corporate Drive, Andover, MA 01810,USA; E-Mails: natalie_twine@eisai.com (N.C.T.); zoltan_dezso@eisai.com (Z.D.); sergei_agoulnik@eisai.com (S.I.A.)

**Keywords:** folate receptor alpha, FRA, ovarian cancer, fallopian tube, 1-carbon metabolism, reduced folate carrier, methylation

## Abstract

Folate receptor alpha (FOLR1/FRA) is reported to be overexpressed in epithelial ovarian cancers (EOC), especially the serous histotype. Further, while dysregulation of the folate-dependent 1-carbon cycle has been implicated in tumorogenesis, little is known relative to the potential mechanism of action of FOLR1 expression in these processes. We therefore investigated the expression of FOLR1, other folate receptors, and genes within the 1-carbon cycle in samples of EOC, normal ovary and fallopian tube on a custom TaqMan Low Density Array. Also included on this array were known markers of EOC such as MSLN, MUC16 and HE4. While few differences were observed in the expression profiles of genes in the 1-carbon cycle, genes previously considered to be overexpressed in EOC (e.g., FOLR1, MSLN, MUC16 and HE4) showed significantly increased expression when comparing EOC to normal ovary. However, when the comparator was changed to normal fallopian tube, these differences were abolished, supporting the hypothesis that EOC derives from fallopian fimbriae and, further, that markers previously considered to be upregulated or overexpressed in EOC are most likely not of ovarian origin, but fallopian in derivation. Our findings therefore support the hypothesis that the cell of origin of EOC is tubal epithelium.

## 1. Introduction

Ovarian cancer causes more deaths than any other cancer of the female reproductive system, with more than 15,000 deaths expected in 2013 in the United States alone [1]. The molecular processes involved in ovarian cancer development are not fully understood, though several risk factors have been described [2,3]. Differential gene expression analyses comparing epithelial ovarian cancer (EOC) to normal ovary tissue identified several potential tumor antigens, including folate receptor 1 (folate receptor alpha; FORL1) which is reported to be overexpressed, especially in serous adenocarcinomas [4]. As such, FOLR1 is considered to be a potential causative agent in tumorogenesis, prompting its extensive investigation as a diagnostic, prognostic, and therapeutic target [5–8].

FRA (folate receptor alpha), the product of the FOLR1 gene, is a glycosylphosphatidylinositol (GPI)-anchored protein that binds plasma folate (5-methyltetrahydrofolate) with high affinity (K_D_ ~ 1 nM), and transports it into the cell via endocytosis [6]. Folate is essential for 1-carbon metabolism, transferring single carbon units in reactions involving purine and pyrimidine synthesis, DNA repair, and methylation of various biomolecules including DNA, proteins, phospholipids, and neurotransmitters [9–12]. Folate deficiency has been linked with dysregulation of these processes which in some cases is associated with an increased risk of developing cancer [6–12].

It was initially thought that epithelial ovarian cancers derive from epithelial cells covering the ovary or those that line inclusion cysts contained within the ovary [2,13]. However, recent studies have shown that most EOCs do not exhibit characteristics typical of mesodermal epithelium from which the ovaries develop and it has been hypothesized that EOCs, particularly those of the serous histotype, originate from the fallopian tubal fimbria in the form of inclusion cysts which may or may not already exist in the cancerous state [14–19]. The tubal epithelium along with other reproductive organs derives from the müllerian duct, which in turn is derived from the mesoderm. Previous work within our laboratory, based on immunohistochemistry expression of FRA, has shown a strong correlation between ovarian serous carcinomas and normal and inflamed fallopian tubes [20]. However, no specific data has been described on the relative expression of the family of folate receptors or, further, genes involved in 1-carbon metabolism or folate homeostasis in normal ovary relative to EOC, to evaluate what role, if any, these may play in tumorogenesis.

Given the hypothesis that EOC derives from fallopian fimbriae, we further sought to examine the expression of known markers of EOC including mesothelin (MSLN) [21–24], mucin 16 (MUC16/CA125) [21,24,25], human epididymis protein 4 (HE4/WFDC2) [23,24,26–28], and epithelial cell adhesion molecule (EPCAM) [29], relative to their expression in normal fallopian tube (FN) and fallopian adenocarcinoma (FA). Additional comparators were normal ovarian tissue (ON) and normal adjacent tissue (NAT) derived from EOC specimens in an effort to more accurately discriminate between potential driver genes or passenger genes relative to the pathogenesis of EOC, employing focused gene expression profile analyses using a custom Taqman Low Density Array (TLDA).

Although some changes in 1-carbon cycle genes were observed, notably in thymidylate synthase, globally this metabolic pathway was relatively unchanged between normal and tumor tissue. On the other hand, we found that the expression differences observed for FOLR1, other known EOC markers (e.g., MSLN, MUC16), and genes involved in signal transduction in EOC were lost when the comparison was changed from normal ovary to normal fallopian tube. These data confirm, or at the very least support the hypothesis that gene expression of EOC correlates well with that of fallopian normal tissue samples and further support the hypothesis that EOC derives from tubal fimbriae. Finally, these data suggest that genes/proteins commonly considered to be markers of EOC are not *a priori* overexpressed in EOC but, rather, their expression is retained from the cell of origin of EOC and should therefore be considered passengers rather than drivers or a result of tumorogenesis *per se*. These findings reveal how application of fallopian tube normal tissue as the tissue of origin may fundamentally change certain paradigms that exist in ovarian cancer gene expression profile studies and ultimately lead to the discovery of new potential therapeutic targets.

## 2. Results and Discussion

### 2.1. Gene Selection

Focused gene expression profiling was conducted as described in Materials and Methods using a custom TLDA (Taqman Low Density Array). The selection criteria used to identify differentially expressed genes between various tissue types consisted of an initial filtering on confidence at *p* ≤ 0.05, followed by filtering on expression level (≥2-fold change).

The focused gene set was comprised of 96 genes (90 genes of interest and 6 controls) subdivided into separate function-related categories. These included folate transporters/receptors, genes involved in 1-carbon cycle metabolism and folate homeostasis, known markers of ovarian cancer and genes involved in signal transduction (Table 1). A number of miscellaneous genes that were shown to directly or indirectly correlate with FOLR1 expression in GeneChip analyses of EOC samples were also included. Genes, classifications and gene descriptions are found in Supplemental Table S1.

### 2.2. Donor Sample Characteristics

The gene panel was used to generate gene expression profiles from 68 EOC, 8 fallopian adenocarcinoma (FA), 9 fallopian normal (FN), 30 EOC NAT and 22 ovarian normal (ON) tissues. Normal tissues were acquired from women who were having other tissues removed for either benign or malignant conditions. Samples were obtained from commercial suppliers representing multiple institutions. Each sample was from an individual donor. Detailed donor demographics are presented in Table 2. Neither age, nor tumor stage showed any significant correlation with differential gene expression (data not shown).

### 2.3. Comparative Gene Expression Profiles in EOC Compared to ON or NAT

At the outset of this study we considered it critical that the correct or most appropriate comparisons be made to more accurately delineate gene expression differences. Since the tissues used in this study were not macrodissected, expression profile differences may be partly attributable to, or confounded by, the heterogeneity of cell types, and relative contributions therefrom, potentially present (e.g., normal, stromal, and tumor) within the tissues collected from multiple donors. Further, several authors have described the potential consequences stemming from field effects whereby adjacent, non-tumorous cells may undergo genetic changes due to their proximity to environment-altering tumorogenic cells [30]. Therefore, the use of NAT, commonly used as the comparator in gene expression studies, may not be optimal when striving to maximize the discriminatory power in gene expression analyses between tumor and non-tumor (normal) tissues, especially if expression differences are low. We therefore assessed the expression of this focused gene set in EOC through comparisons to both ovarian tumor NAT and ON.

**Figure 1:**
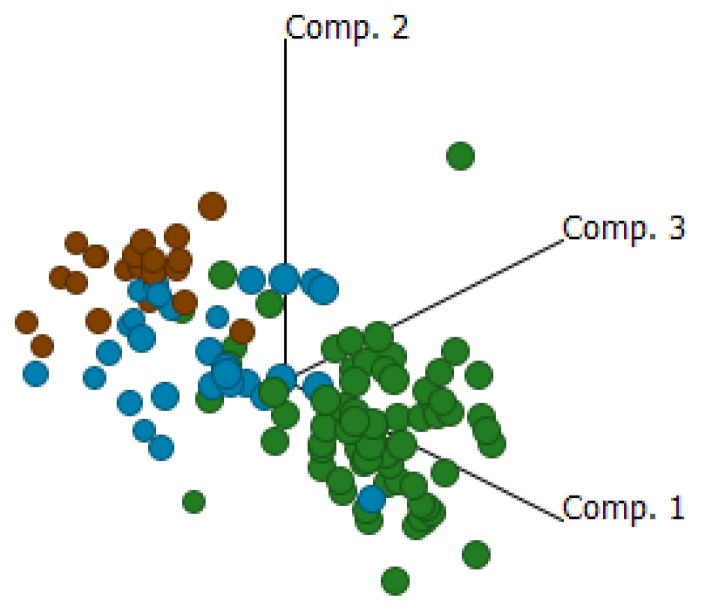
Principle component analysis of the 90 gene set on the custom TLDA comparing samples of ovarian normal (ON; brown), epithelial ovarian cancer (EOC; green) and normal adjacent (ovarian) tissue (NAT; blue).

Comparisons between EOC, NAT and ON are shown in Supplemental Table S2. Figure 1 shows a principle component analysis of all 90 genes investigated in the present study and demonstrates a clear separation between EOC and ON, as expected. It can also be seen that NAT samples are more disperse and primarily lie between samples of EOC and ON, with some samples more normal-like and others more tumor-like. Overall, expression levels for the investigated genes were much higher in NAT than in ON and, as expected, the magnitudes of the fold differences for EOC were significantly reduced when comparison was made to NAT relative to ON. These data support previous findings and strongly suggest that ON rather than NAT should be used in comparative gene expression analyses in EOC, and probably other cancers. It should be noted, however, that expression analyses on NAT may be useful in determining the influence or impact of tumor components other than tumor cells, such as tumor associated macrophages (TAMs) or tumor infiltrating lymphocytes (TILs) on the microenvironment associated with the tumor itself. However, for absolute/discriminatory gene expression profiles, comparisons should be made to normal tissue derived from non-diseased subjects. It can also be seen in Figure 1 that while the clustering of tissue types is clear, there is some dispersion, most notable with several EOC samples clustering closer to normal ovary than other EOC samples. This is most likely a reflection of the variable tumor content in samples since, as noted above, no macro-dissection or other tumor enrichment was performed.

### 2.4. Cluster Analysis Reveals Strong Similarities between EOC and Fallopian Tube (FN)

We recently published findings that 100% of serous ovarian adenocarcinomas analyzed expressed FRA protein and that staining was comparable to that observed on normal fallopian tube epithelial cells [20]. If fallopian tube is indeed the tissue of origin for serous ovarian adenocarcinomas, then EOC gene expression profiles would be expected to more closely resemble that of fallopian tube tissue than normal ovarian tissue.

**Figure 2:**
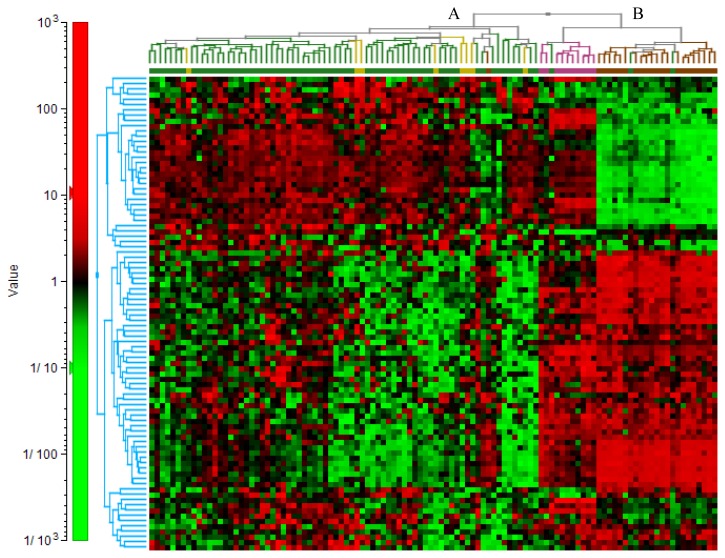
2D hierarchical clustering of the 90 gene set. The samples are displayed on the *x*-axis and the genes are displayed on the *y*-axis. Analysis shows that Cluster A contained all (8/8) of the fallopian adenocarcinoma (FA; yellow) and 65/68 EOC (green) samples. Cluster B contained all (10/10) of the fallopian normal (FN; pink), all of the ON (22/22) (brown) and 3/68 EOC samples.

Comparisons between ON, EOC, FN and FA are shown in Supplemental Table S3. We performed 2D hierarchical cluster analysis based on all 90 genes in our custom TLDA comparing gene expression profiles between ON, EOC, FN, and FA. Results are illustrated in Figure 2 and show that two distinct clusters were identified. Cluster A contained all of the FA and 65 out of 68 EOCs. Cluster B contained all of the FN and ON and 3 out of the 68 ovarian tumors.

**Figure 3:**
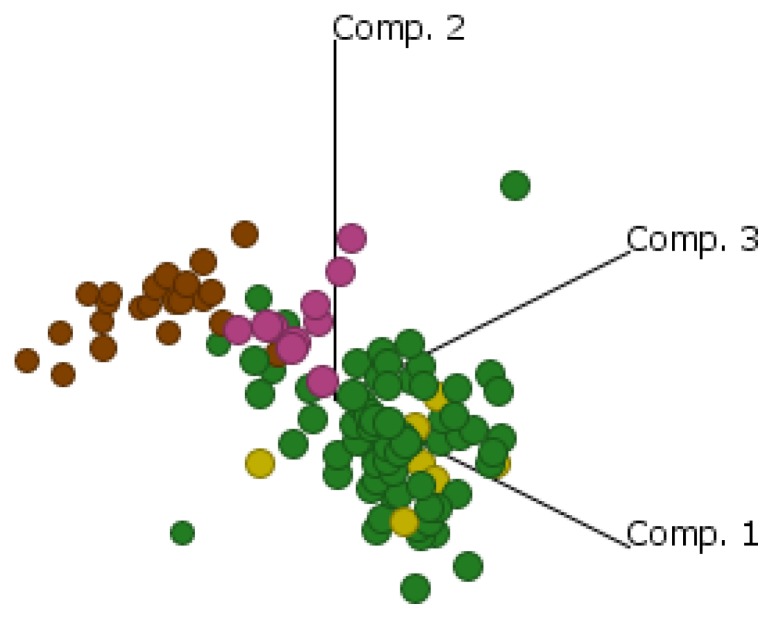
Principle component analysis of all 90 genes: EOC (green) closely clusters with FA (yellow) and separately to ON (brown). FN (pink) clusters somewhat in between EOC and ON.

Comparable results were seen when data were mapped using principal component analysis (Figure 3). EOC clustered closely with FA, which were both distinctly separated from the ON cluster. FN samples were shown to cluster somewhat in between the EOC/FA and ON clusters suggesting that ovarian serous and fallopian adenocarcinoma are more fallopian-like than ovary-like, supporting the hypothesis that EOC develops from fallopian fimbriae. These data also exemplify the need to compare gene expression profiles against the tissue from which the tumor derives, *i.e.*, the cell of origin. Although it should be noted that there were relatively few fallopian tissue samples analyzed compared to EOC samples, we did observe an apparent difference between FN and FA which warrants further study with larger sample sizes.

#### 2.4.1. Folate Transport and Regulation

Folate receptor alpha (FRA/FOLR1) has recently been described to be overexpressed in EOC and is proposed as a specific target in EOC with several drug candidates in clinical trials [6–8]. Further, FRA expression in EOC has been reported to be a result of tumorogenesis since it is not expressed in normal ovary, adding to its viability as a target for therapy. However, more recent data suggesting that EOC derives from the fallopian fimbriae, prompted us to investigate the expression of FRA/FOLR1 and other members of the folate receptor family in fallopian tube relative to EOC.

Indeed, switching the comparative baseline from ON to FN significantly altered the gene expression profile for FOLR1. FOLR1 was upregulated over 100-fold (FC = 113.3, *p* = 2.15 × 10^−16^), when comparing EOC relative to ON, but did not achieve statistical significance (FC = 0.46, *p* = 2.19 × 10^−1^) when compared against FN. This data suggests that the expression of FOLR1 in EOC is a passenger effect, rather than a driver, and further supports the hypothesis that EOC is derived from fallopian tube tissue, *i.e.*, the cell of origin of EOC is the epithelial lining of fallopian fimbriae. Importantly, FOLR1 expression was also unchanged when comparing FN to FA (FC = 0.68; *p* = 6.37 × 10^−1^).

Differences in expression levels of the other folate receptors were not as large relative to that seen for FOLR1. The ubiquitous reduced folate carrier (RFC, SLC46A1) was slightly downregulated in EOC when compared to fallopian tissue (FC = 0.36; *p* = 1.21 × 10^−6^) but this was not significant when compared to ON (FC = 0.58; *p* = 9.15 × 10^−5^). Expression changes for FOLR2 (folate receptor beta) and SLC19A1 (PCFT, proton-coupled folate transporter) in EOC were negligible, independent of the comparator tissue used. FOLR3 FC = 12.96; *p* = 8.02 × 10^−7^) was upregulated using ON, but was less significantly different when compared to fallopian tube tissue (FC = 4.54; *p* = 2.23 × 10^−2^). However, it should be noted that FOLR3 (folate receptor gamma) is reportedly a secreted folate receptor and little is known of its biology or biologic function [31]. FOLR4 (folate receptor delta) was downregulated in EOC when compared to ON (FC = 0.04, *p* = 2.05 × 10^−4^) but was unchanged when the comparison was made to FN (FC = 0.88, *p* = 9.81 × 10^−1^).

**Figure 4:**
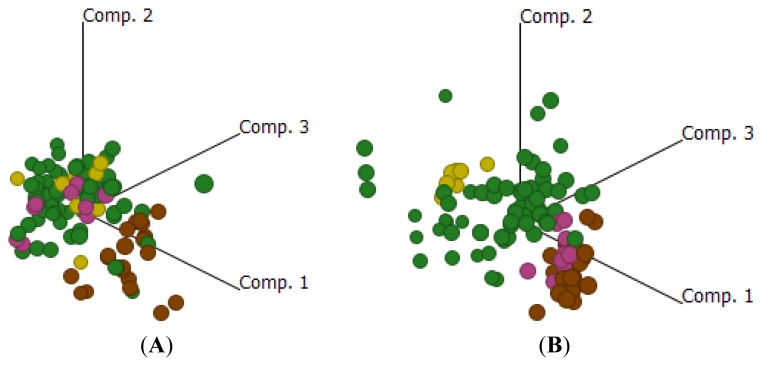
(**A**) Principle component analysis of folate receptor genes relative to ON (brown), EOC (green), FN (pink) and FA (yellow) samples; (**B**) Principle components analysis of 1-carbon cycle metabolic and folate homeostasis genes relative to ON (brown), EOC (green), FN (pink) and FA (yellow).

Comparisons between EOC, ON, FN and FA are shown in Supplemental Table S3. As can be seen from the principal component analysis (Figure 4A), using just the folate receptor data the EOC and fallopian tube samples, both normal and adenocarcinoma, group distinctly from the normal ovarian samples, driven in large part by the expression of FOLR1. A 2D hierarchical analysis is shown in Supplemental Figure S1. As a group therefore, the expression of the folate receptor family shows that EOC is more fallopian-like than ovary-like. Importantly with respect to folate receptor expression and unlike the global expression data shown in Figure 3, there appears to be no distinction between normal or diseased fallopian tube.

#### 2.4.2. One-Carbon Cycle Metabolism, DNA Replication/Repair

Although FRA/FOLR1 is the subject of intense research with respect to drug targeting approaches, especially in EOC, little is known with respect to what effects, if any, the high expression of FOLR1 may have on the 1-carbon cycle. The 1-carbon cycle plays an essential role in mammalian cells, not the least of which is its criticality in the synthesis of purines and pyrimidines and other methylation reactions including DNA methylation, a hallmark of cancer [32,33]. After gaining entry into the cell via one or more members of the folate receptor family, 5-methyltetrahydrofolate is involved in numerous transformations and trans-methylation reactions, terminating on one hand with thymidylate synthase (TYMS) and on the other, with gamma-glutamyl hydrolase (GGH), an enzyme responsible for the de-glutamylation of folates, regulating their retention/secretion from cells [34]. We therefore chose to investigate the relative expression of the various enzymes involved in 1-carbon metabolism and folate homeostasis in the cohort described herein.

Not surprisingly given its role in DNA replication/repair and methylation in tumor cells, TYMS was shown to be upregulated approximately 5-fold in both EOC and fallopian tube tumors when compared to FN or ON. Interestingly, GGH, was also shown to be upregulated in EOC compared to ON or FN. Furthermore, we observed a generalized downregulation of genes associated with the methylation pathway (e.g., BHMT, MTHFR, BHMT2, CTH, MAT1A) in EOC and FA, independent of the baseline used. Downregulation of these genes could influence the balance between methionine, homocysteine, and cysteine levels within tumors cells and may, at least in part, account for global changes in methylation patterns commonly observed across various tumors [32–34].

Comparisons between EOC, ON, FN and FA with respect to genes involved in 1-carbon metabolism and folate homeostasis are shown in Supplemental Table S3. Using 2D hierarchical analysis, 2 clusters were identified (Supplemental Figure S2). Cluster A contained predominately normal tissue (ovarian and fallopian) while cluster B was comprised predominately of tumor samples, both EOC and fallopian adenocarcinoma. This is seen more clearly in principal component analysis (Figure 4B). These distinct clusters observed for the 1-carbon cycle between tumor and normal tissues suggest a global change in the cells requirements for methyl groups (and associated reactions), most likely in support of increased metabolic activity and growth in the tumor. However, the biochemical details of these changes are beyond the scope of this work.

#### 2.4.3. Known Markers of Ovarian Cancer

**Figure 5:**
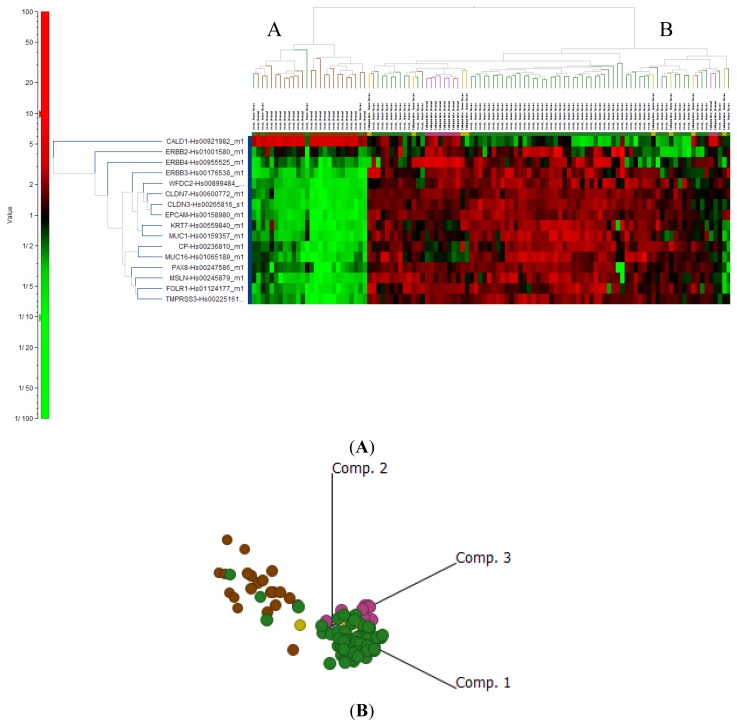
Known markers of ovarian cancer cluster with fallopian tube not normal ovary. (**A**) 2D hierarchical clustering of genes shows 2 distinct clusters: Cluster A contained 4/68 EOC (green) and all (22/22) ON (brown) samples. Cluster B contained 64/68 EOC, all (10/10) FN (pink) and all (8/8) FA (yellow); (**B**) Principle component analysis of ON (brown), EOC (green), FN (pink) and FA (yellow).

In an effort to further define the fallopian tube as the tissue of origin of EOC, we investigated the expression of known markers of EOC, many of which, including CA125 (MUC16), MSLN, HE4, and more recently FOLR1 (FRA), are used diagnostically and/or are being investigated therapeutically. Other genes that have been proposed as potential diagnostic markers for EOC, including PAX8 [35,36] KRT7 [37], CP [38], EPCAM [29], SLC34A2 [39], TMPRSS3 [40] and the ErbB-family of receptors [41] were also evaluated. As expected, these genes were significantly upregulated when EOC samples were compared to ON (Figure 5). However, none of these genes were differentially expressed when the comparison was made to either FN or FA (Supplemental Table S3). Interestingly, CALD-1, which plays an essential role in mitosis and receptor capping and has been proposed as a diagnostic for ovarian cancer, was the only marker studied that was downregulated when compared to ON or FN samples [36].

2D hierarchical analysis (Figure 5A) and principal component analysis (Figure 5B) of these 16 EOC markers showed two very distinct clusters with a clear separation between normal ovary from EOC, normal fallopian tube and fallopian adenocarcinoma, again supporting the fallopian origin of EOC. These data suggest that commonly accepted markers of EOC, such as MSLN, HE4 and CA125 (MUC16), are not derived from the ovary but, as described above for FOLR1 expression, are passengers in the development of EOC from fallopian epithelial cells. This is substantiated by our findings that these genes were also expressed at very low levels in ON when compared to FN. This finding does not diminish the diagnostic or therapeutic potential of these genes/proteins, but does relate to the known “baseline” values seen in the serum of normal patients, *i.e.*, normal expression is seen/expected and increased serum levels reflect the increased “volume” of cells (tumor burden) expressing these proteins.

#### 2.4.4. Signal Transduction Genes

**Figure 6:**
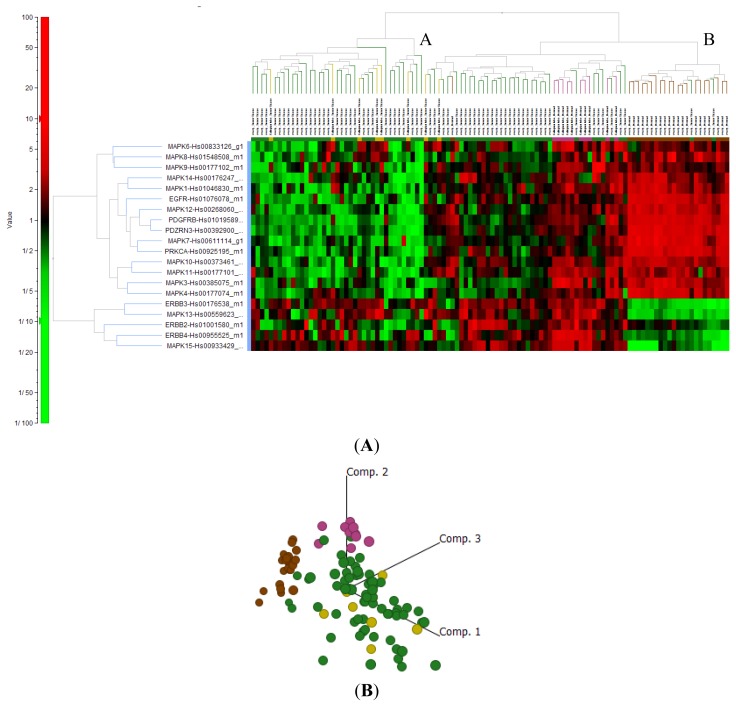
Signal transduction genes show distinct clustering. (**A**) 2D hierarchical clustering of genes shows 2 distinct clusters: Cluster A contained 66/68 EOC (green), all (10/10) FN (pink) and FA (8/8) and 1/22 ON. Cluster B contained 2/68 EOC (green) and 21/22 ON (brown); (**B**) Principle component analysis of ON (brown), EOC (green), FN (pink) and FA (yellow) samples.

Most signal transduction genes investigated in the present study (e.g., PDGFR, MAPK10, MAPK11, MAPK12, MAPK3 and MAPK4) were shown to be significantly downregulated when EOC was compared to ON, and to a lesser degree, FN. Interestingly, there were discrepancies in expression levels in some genes in this subset. For example, a few genes, including ERBB4 and MAPK15, had extremely high expression in EOC when compared to ON, yet were downregulated when compared to FN. Upon further analysis, it appeared that these genes were expressed at very low levels in ON when compared to FN. Furthermore, PRKCA, EGFR and MAPK7 were significantly downregulated when compared to ON but this was not significant when compared to FN. We found that all three of these genes were expressed at higher levels (2–3 fold) in ON when compared to FN (Supplemental Table S3). 2D hierarchical analysis (Figure 6A) showed that Cluster A contained 64 out of 68 EOC, and all FN and FA tissue and that Cluster B contained 20 out of 22 ON tissues. While the principal component analysis (Figure 6B) shows more dispersion of samples compared to that shown in Figure 5 for markers of EOC, the clustering is still quite clear and shows EOC clustering with all fallopian tube cancers and a clear separation from ON.

#### 2.4.5. Steroid Receptors

Given that the expression of FOLR1, and numerous other genes, is at least in part regulated through the action of steroid receptors [42,43], we included an analysis of the estrogen receptor (ESR1), androgen receptor (AR) and the progesterone receptor (PGR) in the current study. Both AR and PGR were significantly downregulated in EOC when compared to FN or ON, as expected [44,45]. Further, there was a significant downregulation of ESR1 when EOC was compared to ON but this was not significant when compared with FN. All three steroid receptors were also downregulated in FA when compared to FN, indicating a possible cancer-related change common to both fallopian adenocarcinoma and EOC (Supplemental Table S3).

## 3. Experimental Section

### 3.1. Sample Description

Fresh frozen samples were obtained from ProteoGenex Inc. Total RNA from fresh frozen samples was obtained from Asterand and Origene. All institutions participating in this study have Institutional Review Board (IRB) approval for human subject research. ON (22) and FN (9) tissue samples were acquired from unrelated donors. 47 EOC and 22 non-cancerous ovaries were obtained from Asterand; 9 FN tissues were purchased from Origene; 21 ovarian tumors, 30 ON adjacent tissues (NAT) and 8 fallopian tube tumors were obtained from ProteoGenex Inc. Sample demographics is presented in Table 2.

### 3.2. RNA Isolation Method and Gene Expression Profiling Using Affymetrix Human Exon Array ST 2.0

Total RNA samples were isolated using TRI Reagent (Sigma, St. Louis, MO, USA) from the obtained fresh frozen tissue and the concentration and quality for all obtained samples were analyzed using Nanodrop and Agilent Bioanalyzer, respectfully. GeneChip analysis was performed on EOC samples to identify genes correlating to FOLR1 expression. The Affymetrix Exon Array ST 1.0 using the Ambion WT Expression kit was used as directed with 100 ng of total RNA. Array data was normalized using standard RMA Refiner workflow in GeneData. A similarity search to find those genes that show high correlation (Positive or Negative) in ovarian samples from Proteogenex was performed in GeneData Analyst using search by distance with a similarity measurement of correlation. A total of 27 genes had +/− correlation greater than 0.8.

### 3.3. Gene Expression Profiling Using Custom Taqman Low Density Array

A real-time qPCR Taqman Low Density Array (TLDA), a 384-Well Micro Fluidic Card, was designed using a focus gene set approach. Genes for the TLDA design were selected based on their role in pathways associated with folate transporters/receptors, one-carbon metabolism and folate homeostasis, known markers of ovarian cancer, signal transduction, steroid receptors and a number of miscellaneous genes (Table 1, Supplemental Table S1). Six endogenous controls were also included.

The TLDA represented these 96 genes in a single reaction, four cDNA samples per plate. Two different methods of cDNA generation were used because of availability of reagents at the time of sample receipt. cDNA was generated from 1 μg of total RNA using High Capacity cDNA Reverse Transcription Kit from Life Technologies for the ovarian samples and SuperScript VILO MasterMix from Life Technologies for the fallopian samples. 100 ng of equivalent cDNA was combined with TaqMan^®^ Gene Expression Master Mix for each port, 2 ports per sample. TLDA was run using standard cycling times on the 7900HT instrument using the autoloader. Raw SDS files from the 7900HT were inspected using the ExpressionSuite Software v1.0. *C*t thresholds were automatically calculated for all TLDA’s for each gene, with the exception of TYMS which was manually set because the software’s algorithm was not able to calculate a proper threshold. All six endogenous controls were used to calculate the Δ*C*t as described in the ExpressionSuite Software. *C*t and Δ*C*t were exported to excel where 2^−Δ^*^C^*^t^ was calculated for each sample [46]. 2^−Δ^*^C^*^t^ was imported into GeneData Analyst for further comparisons and graphs.

Two-dimensional (2D) hierarchical clustering [47] and principal component analysis (PCA) [48] were performed using GeneData Analyst. 2D hierarchical clustering used Manhattan distance and complete linkage. For expression representation of the genes in the 2D clustering, genes were normalized using row scaling, which transforms each row (gene) of the transformed data matrix by subtracting the row mean from each value and dividing the results by a scaling factor derived from the row data. The values are than represented as a ratio above or below the mean of that row. Covariance matrix was used for PCA.

## 4. Conclusions

In the present study we used a custom TLDA to investigate the expression of the folate receptor family, genes of the 1-carbon cycle pathway, known EOC markers and a number of other genes implicated in EOC progression including signal transduction. Comparisons were made to expression levels in normal adjacent tissue (NAT) from EOC patients as well as to normal fallopian tube tissue and fallopian adenocarcinoma samples. Our data clearly shows that NAT is inappropriate as a comparator in EOC gene expression studies with some NAT samples being more normal-like while others more closely resemble tumor. This is likely a generic phenomenon and gene expression studies comparing diseased (tumor) and NAT should be viewed with caution. It should also be noted that since in the present study normal tissues were acquired from women who had benign or other malignant conditions, although not of the tissues in question, these samples may not be totally representative of gene expression in normal fallopian tube or ovary from healthy women but clearly represent a more appropriate comparator in gene expression studies.

While not definitive and worthy of additional study, some changes were noted in the expression of 1-carbon cycle and folate homeostasis genes that suggest alterations in global methylation, indicative of the role of DNA replication/repair and methylation in cancer cell proliferation. Not unexpectedly, TYMS (thymidylate synthase) was shown to be significantly upregulated in both EOC and fallopian adenocarcinoma relative to normal tissues but to approximately the same level when the two cancer types were compared. Perhaps surprisingly, GGH (gamma-glutamyl hydrolase) expression also appeared to be increased in tumor samples *versus* normal tissue. Since GGH is the terminal enzyme responsible for de-glutamylation of folate species and subsequent release from the cell, this may reflect an increased turnover of folates in tumor tissues relative to normal tissues. Indeed, the high expression of FOLR1 may result in increased cellular folates and the increased GGH expression may be a reflection of the balance in the flux of folates into and out of the cell. The very high affinity of FOLR1 for folates, relative to the RFC, is likely advantageous to rapidly growing tumors in ensuring adequate supply of folate for DNA synthesis and methylation reactions.

Finally, we demonstrated that genes previously considered to be overexpressed in EOC (e.g., FOLR1, MSLN, MUC16, MUC1, CLDN3, CLDN7 and HE4) were indeed significantly overexpressed in EOC when compared to normal ovarian tissue. Importantly however, none of these markers of EOC, with the possible exception of MUC16, showed significant upregulation when compared to fallopian tube tissue, either normal tissue or fallopian adenocarcinoma. Cluster analysis produced two very distinct clusters, one of which contained ON tissue and a second cluster that contained normal and cancerous fallopian tissue as well as EOC. Taken together, these data support the hypothesis that EOC derives from fallopian fimbriae and, further, that markers previously considered to be upregulated or overexpressed in EOC are most likely not of ovarian origin, but fallopian in derivation.

## Supplementary Information



## Figures and Tables

**Table 1:** 96-Gene custom Taqman Low Density Array (TLDA) design: Gene categories and sample size (*N*).

Gene Categories	Sample Size
Folate Transporters/Receptors	6
One-Carbon Metabolism and folate homeostasis	19
Known Markers of Ovarian Cancer	13
Signal Transduction	19
Steroid Receptors	3
Miscellaneous Genes	30
*Endogenous Controls*	6

**Total**	**96**

**Table 2:** Demographics and clinical characteristics of cancer and normal sample donors.

Variable	Normal ovary, *N* (%)	Epithelial ovarian cancer, *N* (%)	Fallopian tube normal, *N* (%)	Fallopian tube tumor, *N* (%)
**Tumor histology**				
Normal	22		9	
Normal Adjacent Tissue	30		NA	
Serous Adenocarcinoma		68		8

**Age (Mean, SD)**	62.5, 12.3	57.7, 10.3	52.4, 12.6	60.1, 4.9

**Race**				
White/caucasian	51	68	1	8
Black/African American	1	0	1	0
Native American/Alaskan	0	0	0	0
Unspecified	0	0	7	0

**Tumor grade**^1^,^2^				
Grade 1		17 (25.0)		1 (12.5)
Grade 2		18 (26.4)		0 (0)
Grade 3		23 (33.8)		7 (87.5)
Unspecified		10 (14.7)		0 (0)

**Tumor stage**^1^,^3^				
Stage I		19 (27.9)		3 (17.6)
Stage II		6 (11.7)		1 (5.9)
Stage III		27 (39.7)		4 (23.5)
Stage IV		8 (11.8)		0 (0)

1 Numbers and percentages exclude Normal and Normal Adjacent Tissues;

2 Tumors were graded according to the three tiered grading scheme;

3 Tumor staging was determined using the International Federation of Gynecology and Obstetrics (FIGO) staging system.
