# Supplementary material for: Gene Expression Analyses Support Fallopian Tube Epithelium as the Cell of Origin of Epithelial Ovarian Cancer
Source: Int J Mol Sci. 2013 Jul 1;14(7):13687–703. doi: 10.3390/ijms140713687 (PMC3742211; doi:10.3390/ijms140713687)
Supplement: Supplementary file 1 [file ijms-14-13687-s001.pdf]

Supplementary Information

Figure S1. 2D Hierarchical Clustering as it relates to the 6 folate transporter/receptor genes. Analysis showed that Cluster A contained 27/68 EOCs (green), 21/22 ON (brown), 1/8 FA cells (pink) and all (10/10) FN (yellow). Cluster B contained 41/68 EOC (green), 1/22 ON (brown) and 7/8 FA.

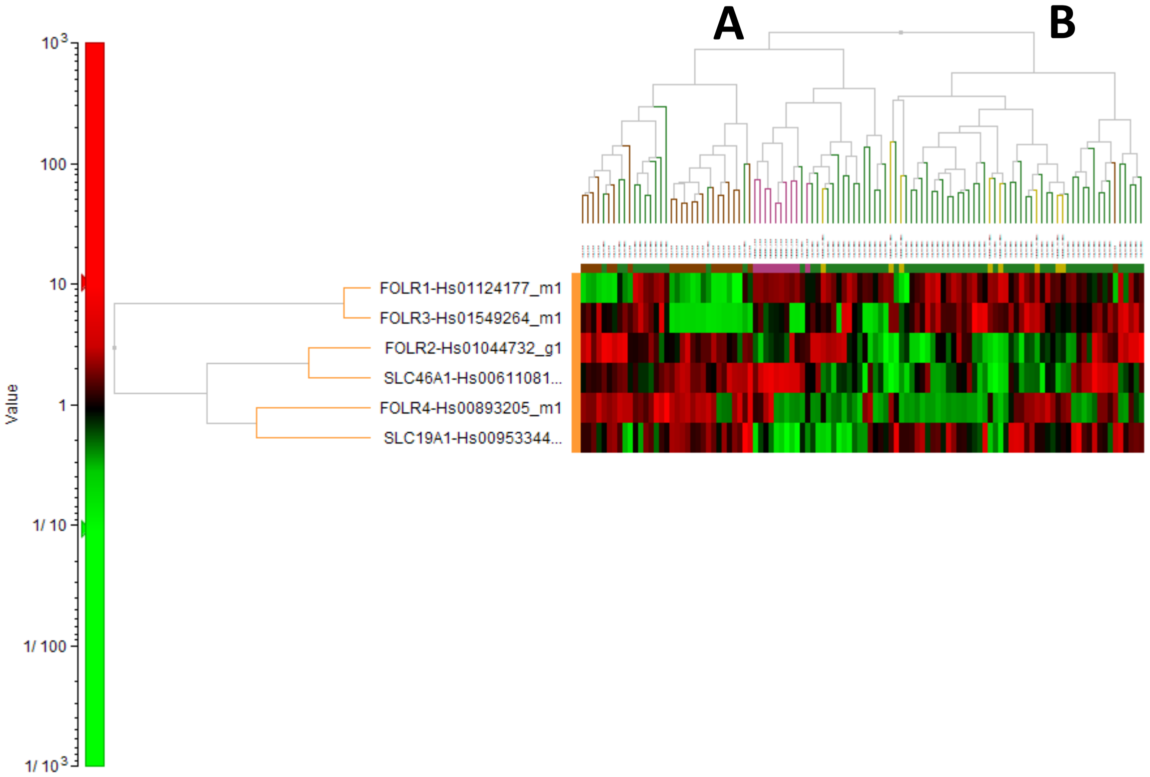

Figure S2. 2D Hierarchical Clustering of genes related to 1-carbon cycle metabolism and folate homeostasis. EOCs cluster more closely with FA tissue than with ON or FN, with regard to the 17 genes involved in 1-carbon cycle metabolism. Two clusters were observed: Cluster A was comprised of 29/68 EOCs, 20/22 ON, 1/8 FA and 10/10 FN; Cluster B was comprised of 39/68 EOCs, 2/22 ON tissues and 7/8 FA.

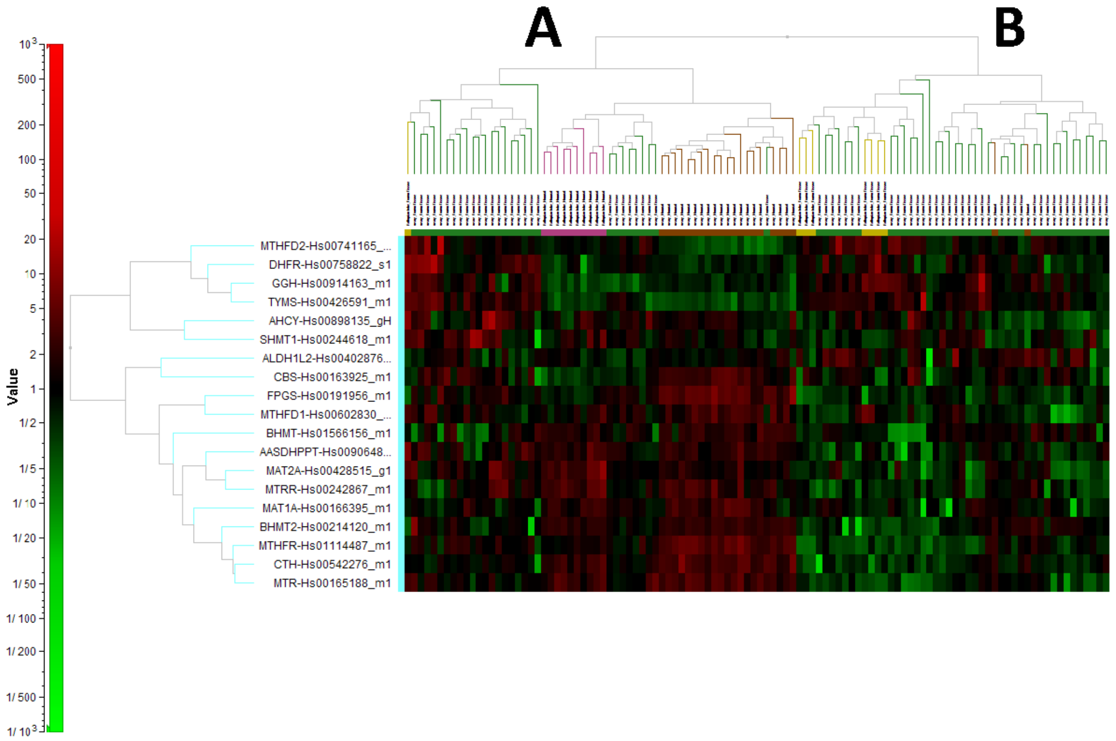

Table S1. Gene Classifications Gene name and Description.

Classification	Gene	Description
**Folate Transporters**	FOLR1-Hs01124177_m1	folate receptor 1 (adult) (FRA)
	FOLR2-Hs01044732_g1	folate receptor 2 (fetal)
	FOLR3-Hs01549264_m1	folate receptor 3 (gamma)
	FOLR4-Hs00893205_m1	folate receptor 4 (delta) homolog (mouse)
	SLC19A1-Hs00953344_m1	solute carrier family 19 (folate transporter) (PCFT)
	SLC46A1-Hs00611081_m1	solute carrier family 46 (folate transporter) (RFC)
**Known EOC Markers**	EPCAM-Hs00158980_m1	epithelial cell adhesion molecule
	CALD1-Hs00921982_m1	caldesmon 1
	CLDN3-Hs00265816_s1	claudin 3
	CLDN7-Hs00600772_m1	claudin 7
	CP-Hs00236810_m1	ceruloplasmin (ferroxidase)
	KRT7-Hs00559840_m1	keratin 7
	MUC16-Hs01065189_m1	mucin 16 (CA-125)
	MUC1-Hs00159357_m1	mucin 1
	PAX8-Hs00247586_m1	paired box 8 [Gene h (CG26667 Celera Annotation)]
	SLC34A2-Hs00197519_m1	solute carrier family 34 (sodium phosphate)
	TMPRSS3-Hs00225161_m1	transmembrane protease
	WFDC2-Hs00899484_m1	WAP four-disulfide core domain 2 (HE4)
	MSLN-Hs00245879_m1	mesothelin

Table S1. *Cont.*

Classification	Gene	Description
	GGH-Hs00914163_m1	gamma-glutamyl hydrolase (conjugase)
	FPGS-Hs00191956_m1	folylpolyglutamate synthase
	AASDHPPT-Hs00906486_m1	aminoadipate-semialdehyde dehydrogenase-phosphopantetheinyl transferase
	AHCY-Hs00898135_gH	adenosylhomocysteinase
	ALDH1L2-Hs00402876_m1	aldehyde dehydrogenase 1 family
	BHMT2-Hs00214120_m1	betaine--homocysteine S-methyltransferase 2
	BHMT-Hs01566156_m1	betaine--homocysteine S-methyltransferase
	CBS-Hs00163925_m1	cystathionine-beta-synthase
**One-Carbon Metabolism and folate homeostasis**	CTH-Hs00542276_m1	cystathionase (cystathionine gamma-lyase)
	DHFR-Hs00758822_s1	dihydrofolate reductase
	MAT1A-Hs00166395_m1	methionine adenosyltransferase I
	MAT2A-Hs00428515_g1	methionine adenosyltransferase II
	MTHFD1-Hs00602830_m1	methylenetetrahydrofolate dehydrogenase (NADP+ dependent) 1
	MTHFD2-Hs00741165_m1	methenyltetrahydrofolate cyclohydrolase
	MTHFR-Hs01114487_m1	methylenetetrahydrofolate reductase (NAD(P)H)
	MTR-Hs00165188_m1	5-methyltetrahydrofolate-homocysteine methyltransferase
	MTRR-Hs00242867_m1	5-methyltetrahydrofolate-homocysteine methyltransferase reductase
	SHMT1-Hs00244618_m1	serine hydroxymethyltransferase 1 (soluble)
	TYMS-Hs00426591_m1	thymidylate synthetase
	ESR1-Hs00174860_m1	estrogen receptor 1
**Steroid Receptors**	AR-Hs00907244_m1	androgen receptor
	PGR-Hs01556707_m1	progesterone receptor
	EGFR-Hs01076078_m1	epidermal growth factor receptor (erb-b1)
	ERBB2-Hs01001580_m1	v-erb-b2 erythroblastic leukemia viral oncogene homolog 2
	ERBB3-Hs00176538_m1	v-erb-b2 erythroblastic leukemia viral oncogene homolog 3 (avian)
	ERBB4-Hs00955525_m1	v-erb-a erythroblastic leukemia viral oncogene homolog 4 (avian)
	MAPK1-Hs01046830_m1	mitogen-activated protein kinase 1
	MAPK3-Hs00385075_m1	mitogen-activated protein kinase 3
	MAPK4-Hs00177074_m1	mitogen-activated protein kinase 4
	MAPK6-Hs00833126_g1	mitogen-activated protein kinase 6
	MAPK7-Hs00611114_g1	mitogen-activated protein kinase 7
**Signal Transduction**	MAPK8-Hs01548508_m1	mitogen-activated protein kinase 8
	MAPK9-Hs00177102_m1	mitogen-activated protein kinase 9
	MAPK10-Hs00373461_m1	mitogen-activated protein kinase 10
	MAPK11-Hs00177101_m1	mitogen-activated protein kinase 11
	MAPK12-Hs00268060_m1	mitogen-activated protein kinase 12
	MAPK13-Hs00559623_m1	mitogen-activated protein kinase 13
	MAPK14-Hs00176247_m1	mitogen-activated protein kinase 14
	MAPK15-Hs00933429_m1	mitogen-activated protein kinase 15
	PDGFRB-Hs01019589_m1	platelet-derived growth factor receptor
	PRKCA-Hs00925195_m1	protein kinase C

Table S1. *Cont.*

Classification	Gene	Description
	ANXA2-Hs00743063_s1	annexin A2
	CPE-Hs00175676_m1	carboxypeptidase E (Gene hCG15254 Celera Annotation)
	DDR1-Hs00233612_m1	discoidin domain receptor tyrosine kinase 1
	DHCR24-Hs00207388_m1	24-dehydrocholesterol reductase
	DSG2-Hs00170071_m1	desmoglein 2
	DST-Hs00156137_m1	dystonin
	GNB2L1-Hs00272002_m1	guanine nucleotide binding protein (G protein)
	JAM3-Hs00230289_m1	junctional adhesion molecule 3
	KIT-Hs00174029_m1	v-kit Hardy-Zuckerman 4 feline sarcoma viral oncogene homolog
	LAPTM4B-Hs00363282_m1	lysosomal protein transmembrane 4 beta
	LSR-Hs00210880_m1	lipolysis stimulated lipoprotein receptor
	MAF-Hs00193519_m1	v-maf musculoaponeurotic fibrosarcoma oncogene homolog (avian)
	RCAN2-Hs00195165_m1	regulator of calcineurin 2
	ST14-Hs01058386_m1	suppression of tumorigenicity 14 (colon carcinoma)
	UCP2-Hs01075227_m1	uncoupling protein 2 (mitochondrial)
**Miscellaneous Genes**	ZEB2-Hs00207691_m1	zinc finger E-box binding homeobox 2
	ARID1A-Hs00195664_m1	AT rich interactive domain 1A (SWI-like)
	ATIC-Hs00269671_m1	5-aminoimidazole-4-carboxamide ribonucleotide formyltransferase/IMP cyclohydrolase
	ERCC1-Hs01012158_m1	excision repair cross-complementing rodent repair deficiency
	NR3C1-Hs00353740_m1	nuclear receptor subfamily 3
	TP53-Hs01034249_m1	tumor protein p53
	TYMP-Hs00157317_m1	thymidine phosphorylase
	PDZRN3-Hs00392900_m1	PDZ domain containing ring finger 3
	ATP7A-Hs00921963_m1	ATPase
	ATP7B-Hs00163739_m1	ATPase
	GCLC-Hs00155249_m1	glutamate-cysteine ligase
	GPX1-Hs00829989_gH	glutathione peroxidase 1
	GSR-Hs00167317_m1	glutathione reductase
	SLC31A1-Hs00977268_g1	solute carrier family 31 (copper transporters)
	SLC31A2-Hs00156984_m1	solute carrier family 31 (copper transporters)

Table S2. Comparisons of gene expression data for EOC *vs*. NAT and EOC *vs*. ON.

Classification	Gene	EOC *vs.* NAT		EOC *vs.* ON	
		p-Value	FC	*p*-Value	FC
**Folate Transporters**	FOLR1-Hs01124177_m1	9.01E-05	7.0019	2.15E-16	113.3125
	FOLR2-Hs01044732_g1	1.59E-04	0.4938	1.49E-02	0.6214
	FOLR3-Hs01549264_m1	3.28E-04	5.4172	8.02E-07	12.9628
	FOLR4-Hs00893205_m1	2.29E-01	0.4009	2.05E-04	0.0411
	SLC19A1-Hs00953344_m1	1.00E-10	2.2532	1.80E-01	0.8491
	SLC46A1-Hs00611081_m1	3.00E-02	0.7413	9.15E-05	0.5821
**Known EOC Markers**	PAX8-Hs00247586_m1	3.69E-06	8.6672	2.78E-13	113.4982
	SLC34A2-Hs00197519_m1	2.80E-06	10.3426	2.79E-16	179.0069
	TMPRSS3-Hs00225161_m1	9.44E-12	30.0421	2.12E-19	91.2426
	CALD1-Hs00921982_m1	5.47E-16	0.2104	8.12E-20	0.1378
	CLDN3-Hs00265816_s1	3.62E-07	6.1418	2.17E-28	103.9800
	CLDN7-Hs00600772_m1	1.32E-09	7.6330	1.57E-26	68.5804
	CP-Hs00236810_m1	7.58E-07	10.9917	4.80E-16	157.7167
	EPCAM-Hs00158980_m1	2.11E-10	7.2228	4.83E-32	68.1680
	KRT7-Hs00559840_m1	1.24E-11	11.2798	1.81E-22	59.1420
	MUC16-Hs01065189_m1	6.81E-12	29.7145	5.18E-21	198.7733
	MUC1-Hs00159357_m1	1.29E-09	5.2751	6.49E-25	32.6951
	WFDC2-Hs00899484_m1	6.90E-05	4.7808	1.51E-22	86.7282
	MSLN-Hs00245879_m1	2.80E-14	14.0035	7.12E-22	34.4299
**One-Carbon Metabolism and folate homeostasis**	FPGS-Hs00191956_m1	3.39E-01	0.9045	1.65E-13	0.3534
	GGH-Hs00914163_m1	1.38E-02	1.5443	1.24E-03	1.9281
	AASDHPPT-Hs00906486_m1	5.57E-03	0.7707	1.62E-04	0.6676
	AHCY-Hs00898135_gH	3.72E-03	1.4038	3.78E-01	0.8908
	ALDH1L2-Hs00402876_m1	5.27E-01	0.8662	1.00E+00	0.9999
	BHMT2-Hs00214120_m1	1.29E-05	0.1802	2.18E-06	0.1179
	BHMT-Hs01566156_m1	1.04E-01	0.4379	4.67E-04	0.1479
	CBS-Hs00163925_m1	3.62E-06	2.8726	3.29E-05	0.4063
	CTH-Hs00542276_m1	1.67E-01	0.7979	4.02E-13	0.2640
	DHFR-Hs00758822_s1	1.55E-04	1.4980	3.03E-03	1.3938
	MAT1A-Hs00166395_m1	2.48E-04	4.3535	1.85E-04	0.2571
	MAT2A-Hs00428515_g1	4.12E-03	1.3049	3.46E-03	0.7440
	MTHFD1-Hs00602830_m1	3.83E-04	1.3665	1.91E-06	0.6259
	MTHFD2-Hs00741165_m1	5.08E-07	2.1005	4.43E-11	3.1702
	MTHFR-Hs01114487_m1	3.44E-01	1.1301	5.17E-14	0.2893
	MTR-Hs00165188_m1	4.14E-01	0.9183	8.03E-11	0.4397
	MTRR-Hs00242867_m1	1.60E-05	0.6883	2.30E-06	0.6270
	SHMT1-Hs00244618_m1	1.75E-03	1.4768	2.85E-01	1.1496
	TYMS-Hs00426591_m1	1.02E-07	3.3592	9.24E-13	6.1374
**Steroid Receptors**	ESR1-Hs00174860_m1	4.79E-03	0.5120	1.66E-01	1.3861
	AR-Hs00907244_m1	1.74E-08	0.2943	1.28E-08	0.2450
	PGR-Hs01556707_m1	1.88E-12	0.0720	2.94E-10	0.0851

Table S2. *Cont.*

Classification	Gene	EOC vs. NAT		EOC vs. ON	
		p-Value	FC	*p*-Value	FC
Signal Transduction	EGFR-Hs01076078_m1	1.18E-06	0.4634	1.78E-12	0.2603
	ERBB2-Hs01001580_m1	6.36E-03	1.4768	3.58E-01	1.1517
	ERBB3-Hs00176538_m1	1.62E-07	3.6412	1.33E-16	10.9879
	ERBB4-Hs00955525_m1	1.17E-04	4.4894	2.20E-07	6.8194
	MAPK1-Hs01046830_m1	7.58E-02	0.8698	3.01E-09	0.5764
	MAPK3-Hs00385075_m1	5.64E-14	0.4502	7.43E-14	0.4253
	MAPK4-Hs00177074_m1	6.72E-08	0.1542	1.57E-13	0.0485
	MAPK6-Hs00833126_g1	1.13E-04	0.6717	1.92E-03	0.7167
	MAPK7-Hs00611114_g1	5.41E-08	0.5523	1.07E-17	0.3236
	MAPK8-Hs01548508_m1	4.68E-02	0.8381	7.92E-07	0.5993
	MAPK9-Hs00177102_m1	3.72E-02	0.8071	1.05E-03	0.6865
	MAPK10-Hs00373461_m1	4.33E-12	0.0937	4.97E-10	0.0929
	MAPK11-Hs00177101_m1	1.69E-07	0.3676	7.29E-07	0.3366
	MAPK12-Hs00268060_m1	1.23E-08	0.3534	2.44E-14	0.1914
	MAPK13-Hs00559623_m1	4.11E-08	2.5215	5.15E-12	3.0565
	MAPK14-Hs00176247_m1	8.60E-01	0.9838	5.33E-07	0.5901
	MAPK15-Hs00933429_m1	1.97E-03	3.7053	2.04E-15	50.8959
	PDGFRB-Hs01019589_m1	2.94E-10	0.1955	5.77E-16	0.0771
	PRKCA-Hs00925195_m1	5.42E-07	0.4061	1.89E-15	0.1755
Miscellaneous Genes	ANXA2-Hs00743063_s1	1.62E-02	0.7118	1.29E-03	1.6144
	CPE-Hs00175676_m1	2.03E-05	0.4475	4.85E-06	0.3874
	DDR1-Hs00233612_m1	1.16E-11	3.7417	1.60E-05	1.9837
	DHCR24-Hs00207388_m1	5.34E-10	3.5317	4.13E-06	2.5238
	DSG2-Hs00170071_m1	7.79E-12	3.0114	9.01E-03	1.3759
	DST-Hs00156137_m1	3.76E-05	0.3849	5.83E-13	0.1332
	GNB2L1-Hs00272002_m1	8.46E-05	0.6317	1.64E-12	0.3575
	JAM3-Hs00230289_m1	1.39E-12	0.1693	1.56E-17	0.0761
	KIT-Hs00174029_m1	2.16E-11	0.1095	4.79E-18	0.0279
	LAPTM4B-Hs00363282_m1	5.31E-10	2.5935	1.56E-06	2.1198
	MAF-Hs00193519_m1	1.21E-10	0.2251	2.01E-12	0.1603
	PDZRN3-Hs00392900_m1	5.96E-11	0.1464	8.27E-18	0.0444
	RCAN2-Hs00195165_m1	2.67E-14	0.1812	1.84E-18	0.1002
	ST14-Hs01058386_m1	6.53E-10	4.6747	5.53E-27	44.0217
	UCP2-Hs01075227_m1	1.09E-06	2.4918	6.92E-23	8.9802
	ZEB2-Hs00207691_m1	5.91E-13	0.2116	6.01E-19	0.0984
	ARID1A-Hs00195664_m1	8.34E-01	1.0211	6.47E-06	0.5879
	ATIC-Hs00269671_m1	4.54E-04	1.3307	8.47E-02	0.8611
	ERCC1-Hs01012158_m1	7.07E-06	0.6392	3.65E-10	0.4728
	NR3C1-Hs00353740_m1	2.17E-08	0.4692	2.47E-09	0.4050
	TP53-Hs01034249_m1	7.73E-01	0.9528	6.51E-02	0.7023
	TYMP-Hs00157317_m1	1.88E-04	2.1127	7.07E-05	2.4232
	LSR-Hs00210880_m1	9.68E-12	3.5118	2.39E-26	11.7775
	ATP7A-Hs00921963_m1	1.02E-01	0.8265	6.94E-08	0.4888
	ATP7B-Hs00163739_m1	1.54E-01	1.2272	2.41E-02	0.7026
	GCLC-Hs00155249_m1	7.89E-05	0.6462	8.84E-01	1.0144
	GPX1-Hs00829989_gH	8.66E-02	0.8759	2.32E-08	1.6489
	GSR-Hs00167317_m1	2.40E-01	0.8860	3.46E-10	0.4680
	SLC31A1-Hs00977268_g1	3.04E-01	0.9136	4.98E-01	1.0654
	SLC31A2-Hs00156984_m1	1.25E-03	0.6432	3.59E-03	0.6353

Table S3. Comparisons of Gene Expression Data for EOC *vs.* FN, EOC *vs.* ON, EOC *vs.* FT, FT *vs.* FN and FT *vs.* ON.

Classification	Gene	EOC *vs.* FN		EOC *vs.* ON		EOC *vs.* FT		FT *vs.* FN		FT *vs.* ON	
		p-Value	FC	*p*-Value	FC	*p*-Value	FC	*p*-Value	FC	*p*-Value	FC
Folate Transporters	FOLR1-Hs01124177_m1	2.19E-01	0.4579	2.15E-16	113.3125	6.04E-01	0.6766	6.37E-01	0.6768	5.61E-07	167.4686
	FOLR2-Hs01044732_g1	6.22E-01	1.1479	1.49E-02	0.6214	1.06E-01	1.6704	1.89E-01	0.6872	7.91E-05	0.3720
	FOLR3-Hs01549264_m1	2.23E-02	4.5434	8.02E-07	12.9628	8.79E-01	1.1137	5.71E-02	4.0794	3.15E-03	11.6391
	FOLR4-Hs00893205_m1	9.18E-01	0.8814	2.05E-04	0.0411	4.84E-01	0.3910	5.87E-01	2.2542	3.36E-02	0.1051
	SLC19A1-Hs00953344_m1	5.14E-06	2.4064	1.80E-01	0.8491	8.67E-02	1.4043	4.30E-02	1.7136	5.08E-03	0.6046
	SLC46A1-Hs00611081_m1	1.21E-06	0.3627	9.15E-05	0.5821	4.67E-04	2.2327	8.38E-07	0.1624	1.69E-08	0.2607
Known EOC Markers	EPCAM-Hs00158980_m1	8.63E-02	1.6764	4.83E-32	68.1680	4.64E-01	0.7861	8.51E-03	2.1324	1.22E-12	86.7113
	CALD1-Hs00921982_m1	3.00E-07	0.2388	8.12E-20	0.1378	3.33E-01	0.7553	1.09E-03	0.3162	2.62E-11	0.1825
	CLDN3-Hs00265816_s1	4.29E-01	1.3699	2.17E-28	103.9800	1.82E-01	1.8277	4.95E-01	0.7495	8.49E-11	56.8909
	CLDN7-Hs00600772_m1	4.83E-01	0.8098	1.57E-26	68.5804	3.29E-01	1.3841	5.68E-02	0.5851	3.53E-07	49.5500
	CP-Hs00236810_m1	1.63E-02	4.6234	4.80E-16	157.7167	5.63E-01	0.6576	1.11E-02	7.0305	4.73E-06	239.8285
	KRT7-Hs00559840_m1	1.66E-02	2.6181	1.81E-22	59.1420	7.00E-01	1.1918	9.63E-02	2.1969	1.73E-07	49.6259
	MUC16-Hs01065189_m1	1.48E-05	10.3402	5.18E-21	198.7733	2.90E-02	3.7759	1.01E-01	2.7384	8.15E-05	52.6420
	MUC1-Hs00159357_m1	6.05E-03	2.5524	6.49E-25	32.6951	5.52E-04	3.8533	2.46E-01	0.6624	1.80E-06	8.4850
	PAX8-Hs00247586_m1	8.91E-01	0.9140	2.78E-13	113.4982	5.36E-01	1.5759	2.03E-01	0.5800	2.76E-04	72.0223
	SLC34A2-Hs00197519_m1	3.14E-01	0.5304	2.79E-16	179.0069	8.45E-01	1.1499	8.25E-02	0.4613	8.89E-06	155.6780
	TMPRSS3-Hs00225161_m1	5.56E-01	0.7762	2.12E-19	91.2426	9.81E-01	0.9886	4.01E-01	0.7852	4.87E-06	92.2954
	WFDC2-Hs00899484_m1	4.51E-01	0.7046	1.51E-22	86.7282	5.46E-01	1.3654	7.63E-02	0.5160	9.10E-10	63.5179
	MSLN-Hs00245879_m1	5.09E-01	1.2877	7.12E-22	34.4299	2.25E-01	1.7278	5.62E-01	0.7453	2.63E-07	19.9272

Table S3. *Cont.*

Classification	Gene	EOC *vs.* FN		EOC *vs.* ON		EOC *vs.* FT		FT *vs.* FN		FT *vs.* ON	
		p-Value	FC	*p*-Value	FC	*p*-Value	FC	*p*-Value	FC	*p*-Value	FC
One-Carbon Metabolism and folate homeostasis	GGH-Hs00914163_m1	6.78E-05	3.3449	1.24E-03	1.9281	1.77E-02	0.4483	6.16E-06	7.4613	8.87E-07	4.3009
	FPGS-Hs00191956_m1	3.27E-01	1.1757	1.65E-13	0.3534	1.76E-05	2.3949	2.92E-03	0.4909	9.09E-11	0.1476
	AASDHPPT-Hs00906486_m1	1.05E-03	0.6023	1.62E-04	0.6676	4.81E-01	0.8842	3.70E-02	0.6811	3.75E-02	0.7550
	AHCY-Hs00898135_gH	5.01E-01	0.8799	3.78E-01	0.8908	8.07E-01	0.9488	7.61E-01	0.9274	7.33E-01	0.9389
	ALDH1L2-Hs00402876_m1	8.49E-01	1.0737	1.00E+00	0.9999	5.53E-01	1.2898	6.07E-01	0.8325	3.76E-01	0.7752
	BHMT2-Hs00214120_m1	2.98E-03	0.1467	2.18E-06	0.1179	1.73E-02	5.5366	2.51E-10	0.0265	1.83E-18	0.0213
	BHMT-Hs01566156_m1	1.52E-02	0.1522	4.67E-04	0.1479	5.90E-01	1.5814	9.32E-07	0.0962	6.13E-06	0.0936
	CBS-Hs00163925_m1	7.78E-03	2.2364	3.29E-05	0.4063	1.20E-01	1.7309	5.10E-01	1.2920	4.40E-05	0.2348
	CTH-Hs00542276_m1	8.17E-06	0.3372	4.02E-13	0.2640	5.76E-02	1.6601	1.26E-05	0.2031	3.67E-10	0.1590
	DHFR-Hs00758822_s1	3.93E-03	1.6371	3.03E-03	1.3938	1.38E-02	0.6318	3.54E-04	2.5913	3.20E-07	2.2062
	MAT1A-Hs00166395_m1	3.19E-03	0.2285	1.85E-04	0.2571	7.15E-01	1.2184	6.43E-06	0.1875	5.41E-04	0.2110
	MAT2A-Hs00428515_g1	1.05E-05	0.5050	3.46E-03	0.7440	7.42E-01	0.9479	1.31E-04	0.5327	1.46E-02	0.7849
	MTHFD1-Hs00602830_m1	6.35E-02	0.7835	1.91E-06	0.6259	6.78E-02	0.7562	8.47E-01	1.0360	2.07E-01	0.8276
	MTHFD2-Hs00741165_m1	4.50E-02	1.5613	4.43E-11	3.1702	1.39E-01	0.6870	9.08E-04	2.2728	6.09E-09	4.6149
	MTHFR-Hs01114487_m1	4.27E-02	0.6644	5.17E-14	0.2893	1.29E-03	2.1361	4.48E-06	0.3110	4.32E-13	0.1354
	MTR-Hs00165188_m1	1.38E-05	0.4711	8.03E-11	0.4397	1.85E-01	1.2844	6.80E-05	0.3668	2.51E-08	0.3423
	MTRR-Hs00242867_m1	2.02E-06	0.5106	2.30E-06	0.6270	7.73E-01	1.0433	1.97E-06	0.4894	4.84E-05	0.6010
	SHMT1-Hs00244618_m1	4.80E-01	0.8741	2.85E-01	1.1496	5.32E-02	1.5059	2.66E-02	0.5805	1.09E-01	0.7634
	TYMS-Hs00426591_m1	1.19E-06	5.1729	9.24E-13	6.1374	8.05E-01	0.9166	1.31E-05	5.6437	3.62E-08	6.6960

Table S3. *Cont.*

Classification	Gene	EOC *vs.* FN		EOC *vs.* ON		EOC *vs.* FT		FT *vs.* FN		FT *vs.* ON	
		p-Value	FC	*p*-Value	FC	*p*-Value	FC	*p*-Value	FC	*p*-Value	FC
Signal Transduction	EGFR-Hs01076078_m1	3.19E-02	0.5903	1.78E-12	0.2603	1.56E-02	2.0296	4.73E-04	0.2908	4.76E-11	0.1283
	ERBB2-Hs01001580_m1	1.51E-01	0.7219	3.58E-01	1.1517	2.71E-01	1.3223	3.25E-04	0.5460	2.25E-01	0.8710
	ERBB3-Hs00176538_m1	3.27E-01	0.7206	1.33E-16	10.9879	6.06E-01	0.8243	6.16E-01	0.8742	3.30E-10	13.3308
	ERBB4-Hs00955525_m1	2.66E-04	0.1541	2.20E-07	6.8194	3.96E-01	1.6272	1.49E-03	0.0947	6.65E-03	4.1908
	MAPK1-Hs01046830_m1	3.14E-04	0.6288	3.01E-09	0.5764	9.40E-01	1.0108	1.28E-03	0.6220	5.75E-07	0.5702
	MAPK3-Hs00385075_m1	6.31E-10	0.3656	7.43E-14	0.4253	9.62E-01	0.9926	4.55E-06	0.3683	5.53E-09	0.4284
	MAPK4-Hs00177074_m1	7.51E-05	0.1221	1.57E-13	0.0485	6.56E-01	0.7725	2.83E-03	0.1581	3.46E-07	0.0628
	MAPK6-Hs00833126_g1	4.94E-05	0.5130	1.92E-03	0.7167	6.84E-03	0.6174	2.26E-01	0.8309	1.25E-01	1.1608
	MAPK7-Hs00611114_g1	3.60E-02	0.7201	1.07E-17	0.3236	2.70E-03	1.7364	4.38E-04	0.4147	1.87E-12	0.1864
	MAPK8-Hs01548508_m1	2.87E-01	0.8618	7.92E-07	0.5993	7.94E-01	1.0427	2.90E-01	0.8265	3.00E-04	0.5748
	MAPK9-Hs00177102_m1	1.15E-02	0.6547	1.05E-03	0.6865	6.04E-01	0.9042	1.22E-01	0.7240	5.42E-02	0.7593
	MAPK10-Hs00373461_m1	5.35E-05	0.1160	4.97E-10	0.0929	7.27E-01	1.2260	6.11E-05	0.0946	2.57E-09	0.0758
	MAPK11-Hs00177101_m1	4.75E-05	0.2826	7.29E-07	0.3366	3.69E-01	0.7389	2.61E-04	0.3825	7.34E-04	0.4556
	MAPK12-Hs00268060_m1	6.05E-04	0.3832	2.44E-14	0.1914	5.30E-02	1.9212	1.64E-03	0.1995	9.35E-09	0.0996
	MAPK13-Hs00559623_m1	5.24E-02	0.6677	5.15E-12	3.0565	1.36E-01	0.6904	9.21E-01	0.9671	4.77E-07	4.4272
	MAPK14-Hs00176247_m1	8.70E-02	0.7763	5.33E-07	0.5901	9.14E-01	0.9824	1.55E-01	0.7903	1.39E-05	0.6007
	MAPK15-Hs00933429_m1	1.36E-05	0.1234	2.04E-15	50.8959	5.19E-01	1.3905	7.68E-05	0.0887	2.92E-04	36.6029
	PDGFRB-Hs01019589_m1	6.57E-05	0.1962	5.77E-16	0.0771	5.84E-01	1.2793	2.48E-04	0.1534	1.43E-11	0.0603
	PRKCA-Hs00925195_m1	1.82E-02	0.5258	1.89E-15	0.1755	1.76E-01	1.5146	8.01E-04	0.3471	1.41E-12	0.1159
Steroid Receptors	ESR1-Hs00174860_m1	2.43E-05	0.2162	1.66E-01	1.3861	5.96E-01	1.2469	2.10E-03	0.1734	7.59E-01	1.1116
	AR-Hs00907244_m1	3.89E-09	0.1117	1.28E-08	0.2450	1.31E-02	2.8602	3.04E-05	0.0391	6.52E-07	0.0857
	PGR-Hs01556707_m1	2.12E-11	0.0182	2.94E-10	0.0851	1.80E-01	2.3001	8.05E-06	0.0079	5.84E-07	0.0370

Table S3. *Cont.*

Classification	Gene	EOC *vs.* FN		EOC *vs.* ON		EOC *vs.* FT		FT *vs.* FN		FT *vs.* ON	
		p-Value	FC	*p*-Value	FC	*p*-Value	FC	*p*-Value	FC	*p*-Value	FC
Miscellaneous Genes	ANXA2-Hs00743063_s1	1.90E-01	1.3175	1.29E-03	1.6144	3.10E-01	1.2799	8.89E-01	1.0294	1.72E-01	1.2614
	CPE-Hs00175676_m1	6.92E-03	0.4598	4.85E-06	0.3874	6.33E-01	1.1642	9.17E-03	0.3950	1.64E-04	0.3328
	DDR1-Hs00233612_m1	1.02E-03	0.4650	1.60E-05	1.9837	4.38E-01	0.8247	2.95E-02	0.5639	2.92E-06	2.4054
	DHCR24-Hs00207388_m1	5.48E-02	0.6126	4.13E-06	2.5238	2.04E-01	0.6897	7.18E-01	0.8882	4.53E-04	3.6594
	DSG2-Hs00170071_m1	3.07E-01	1.2040	9.01E-03	1.3759	1.52E-01	1.3492	6.83E-01	0.8924	9.08E-01	1.0198
	DST-Hs00156137_m1	1.38E-02	0.4026	5.83E-13	0.1332	1.97E-01	1.6958	9.15E-04	0.2374	1.40E-13	0.0786
	GNB2L1-Hs00272002_m1	6.21E-02	0.7128	1.64E-12	0.3575	4.21E-01	1.1866	5.36E-02	0.6007	8.38E-07	0.3013
	JAM3-Hs00230289_m1	7.55E-05	0.2204	1.56E-17	0.0761	6.59E-02	2.1906	2.18E-05	0.1006	4.22E-14	0.0347
	KIT-Hs00174029_m1	7.08E-04	0.1875	4.79E-18	0.0279	5.67E-01	0.7206	4.48E-02	0.2602	1.27E-07	0.0388
	LAPTM4B-Hs00363282_m1	4.01E-02	1.5588	1.56E-06	2.1198	6.20E-03	0.4968	2.10E-04	3.1376	7.89E-09	4.2669
	LSR-Hs00210880_m1	3.00E-01	1.2794	2.39E-26	11.7775	3.96E-01	1.2464	8.96E-01	1.0265	4.96E-14	9.4494
	MAF-Hs00193519_m1	2.91E-01	0.7031	2.01E-12	0.1603	1.39E-03	3.5185	1.88E-05	0.1998	7.36E-16	0.0455
	RCAN2-Hs00195165_m1	1.97E-05	0.2496	1.84E-18	0.1002	8.53E-02	1.8387	2.40E-05	0.1357	9.39E-13	0.0545
	ST14-Hs01058386_m1	3.13E-01	1.2906	5.53E-27	44.0217	3.07E-01	1.3330	8.86E-01	0.9682	5.47E-07	33.0257
	UCP2-Hs01075227_m1	6.08E-04	0.4455	6.92E-23	8.9802	2.33E-02	0.5565	4.53E-01	0.8006	3.43E-11	16.1383
	ZEB2-Hs00207691_m1	1.82E-06	0.2057	6.01E-19	0.0984	6.64E-01	1.1638	1.74E-05	0.1768	1.95E-14	0.0846
	ARID1A-Hs00195664_m1	7.71E-04	0.5676	6.47E-06	0.5879	1.15E-01	1.3610	1.19E-03	0.4170	1.24E-05	0.4320
	ATIC-Hs00269671_m1	8.14E-01	1.0300	8.47E-02	0.8611	4.41E-01	1.1185	6.27E-01	0.9208	3.86E-02	0.7699
	ERCC1-Hs01012158_m1	1.13E-01	0.7796	3.65E-10	0.4728	2.81E-02	1.5258	6.90E-03	0.5110	2.22E-08	0.3098
	NR3C1-Hs00353740_m1	5.41E-06	0.3753	2.47E-09	0.4050	8.07E-01	1.0571	2.36E-05	0.3550	4.17E-08	0.3832
	TP53-Hs01034249_m1	4.67E-02	0.5661	6.51E-02	0.7023	2.24E-01	1.4974	6.04E-03	0.3780	7.74E-04	0.4690
	TYMP-Hs00157317_m1	7.33E-01	0.9089	7.07E-05	2.4232	3.43E-01	1.3546	1.23E-01	0.6710	9.38E-02	1.7889
	PDZRN3-Hs00392900_m1	1.70E-03	0.2481	8.27E-18	0.0444	3.47E-01	1.5891	6.57E-04	0.1561	7.15E-13	0.0279
	ATP7A-Hs00921963_m1	9.78E-07	0.3849	6.94E-08	0.4888	6.62E-01	0.9145	8.25E-05	0.4209	1.18E-05	0.5345
	ATP7B-Hs00163739_m1	1.45E-03	0.4723	2.41E-02	0.7026	8.90E-01	1.0408	6.80E-02	0.4538	1.58E-01	0.6751
	GCLC-Hs00155249_m1	1.26E-15	0.1616	8.84E-01	1.0144	4.06E-01	1.1489	2.14E-04	0.1407	4.47E-01	0.8829
	GPX1-Hs00829989_gH	1.00E-35	18.5022	2.32E-08	1.6489	7.64E-29	11.2603	1.48E-03	1.6431	2.57E-16	0.1464
	GSR-Hs00167317_m1	5.67E-05	0.5119	3.46E-10	0.4680	2.32E-01	0.8082	2.97E-03	0.6334	1.22E-05	0.5790
	SLC31A1-Hs00977268_g1	1.85E-04	0.5784	4.98E-01	1.0654	3.48E-04	0.5487	7.52E-01	1.0541	4.78E-07	1.9418
	SLC31A2-Hs00156984_m1	6.62E-03	0.5456	3.59E-03	0.6353	5.20E-01	0.8504	3.80E-02	0.6415	1.11E-01	0.7471
